# Heterogeneity in the Course of Suicidal Ideation and its Relation to Suicide Attempts in First-Episode Psychosis: A 5-Year Prospective Study

**DOI:** 10.1177/07067437231167387

**Published:** 2023-04-18

**Authors:** Roxanne Sicotte, Srividya N. Iyer, Éric Lacourse, Jean R. Séguin, Amal Abdel-Baki

**Affiliations:** 1 Research Center of the Centre Hospitalier de l'Université de Montréal (CRCHUM), Montréal, Québec, Canada; 2 Department of Psychiatry and Addictology, Faculty of Medicine, Université de Montréal, Montréal, Québec, Canada; 3 Department of Psychiatry, Faculty of Medicine and Health Sciences, McGill University, Montréal, Québec, Canada; 4 Prevention and Early Intervention Program for Psychosis (PEPP), 26632 Douglas Mental Health University Institute, Verdun, Québec, Canada; 5 Department of Sociology, Faculty of Arts and Sciences, Université de Montréal, Montréal, Québec, Canada; 6 Centre Hospitalier Universitaire (CHU) Sainte-Justine Research Center, Montréal, Québec, Canada

**Keywords:** psychotic disorders, first-episode psychosis, suicide, suicidal ideation, suicide attempts, longitudinal studies, trajectories

## Abstract

**Objectives:**

Although the risk of suicide is high in first-episode psychosis (FEP), little is known about the course of suicidal ideation and its relation to suicide attempts. Therefore, we aimed to identify 5-year trajectories of suicidal ideation and associated factors in FEP and compare how suicide attempts were distributed across these identified trajectories.

**Method:**

This 5-year prospective study assessed suicidal ideation, suicide attempts and potentially associated factors through research interviews, chart review and coroners’ reports in 382 FEP patients [mean age = 23.53 (*SD* = 3.61)] admitted to 2 5-year early psychosis services in Montreal, Canada. Trajectories were identified using a semiparametric mixture model, and associated factors with multinomial logistic regression.

**Results:**

Three suicidal ideation trajectories were identified: *low and decreasing* (*n* = 325, 85.08%); *early decline, then increasing* (*n* = 30, 7.85%), and *persistent suicidal ideation* (*n* = 27, 7.07%). Suicidal ideation prior to admission (OR = 2.85, 95% CI, 1.23 to 6.63, *P *< 0.05) and cocaine use disorder (OR = 6.78, 95% CI, 1.08 to 42.75, *P *< 0.05) were associated with the *early decline, then increasing suicidal ideation* trajectory. Persons with prior suicide ideation (OR = 4.33, 95% CI, 1.66 to 11.29, *P *< 0.05) and attempts (OR = 8.18, 95% CI, 2.39 to 27.97, *P *< 0.001) and alcohol use disorder (OR = 3.63, 95% CI, 1.4 to 9.42, *P *< 0.05) were more likely to belong to the *persistent suicidal ideation* trajectory, and to attempt suicide during follow-up.

**Conclusions:**

Our study highlights heterogeneity in the course of suicidal ideation over 5 years and the importance of ongoing assessment of suicidal risk in FEP patients, particularly for patients who persistently report suicidal ideation, as they are likelier to engage in suicide attempts. Patients with factors associated with increasing or persistent suicidal ideation trajectories should be targeted for suicide prevention interventions from the early phase of follow-up. Given the small number of persons in these trajectories and the wide CIs for some factors, larger studies are however needed to further characterize who belongs in each group.

## Résumé

**Objectifs:**

Bien que le risque de suicide soit élevé lors du premier épisode de psychose (PEP), nous en savons peu sur l'évolution des idées suicidaires et son association avec les tentatives de suicide. Par conséquent, notre objectif était d'identifier les trajectoires des idées suicidaires sur une période de cinq ans et les facteurs qui y sont associés chez les personnes présentant un PEP, et de comparer la distribution des tentatives de suicide à travers les trajectoires identifiées.

**Méthode:**

Cette étude prospective de cinq ans a évalué les idées suicidaires, les tentatives de suicide et les facteurs potentiellement associés à partir d’entrevues de recherche, de la révisiondes dossiers médicaux et des rapports du coroner chez 382 patients présentant un PEP [âge moyen = 23,53 (ET = 3,61)], admis dans 2 services d'intervention précoce pour la psychose offrant un suivi de 5 ans à Montréal, Canada. Les trajectoires ont été identifiées avecun modèle mixte semi-paramétrique, et les facteurs associés ont été identifiés avec une régression logistique multinomiale.

**Résultats:**

Trois trajectoires d’idées suicidaires ont été identifiées : *idées suicidaires faibles et décroissantes* (n = 325, 85,08 %); *déclin précoce, puis augmentation des idées suicidaires* (n = 30, 7,85 %), et *idées suicidaires persistantes* (n = 27, 7,07 %). Les idées suicidaires avant l’admission (RC = 2,85, IC à 95 % 1,23 à 6.63, p < 0,05) et le trouble d’usage de cocaïne (RC = 6,78, IC à 95 % 1,08 à 42,75, p < 0,05) étaient associés à la trajectoire de *déclin précoce, puis d'augmentation des idées suicidaires*. Les personnes ayant des antécédents d'idées suicidaires (RC =4,33, IC à 95 % 1,66 à 11,29, p < 0,05) et de tentatives de suicide (RC = 8,18, IC à 95 % 2,39 à 27,97 p < 0,001) ainsi que celles ayantun trouble d'usage de l’alcool (RC = 3,63, IC à 95 % 1,4 à 9,42, p < 0,05) étaient plus susceptibles de faire partie de la trajectoire d’idées suicidaires persistantes, et de faire une tentative de suicide au cours dusuivi.

**Conclusions:**

Notre étude souligne l’hétérogénéité de l'évolution des idées suicidaires sur une période de cinq ans et l’importance d’évaluer de façon continue le risque suicidaire chez les patients ayant un PEP, particulièrement chez ceux ayant des idéessuicidaires persistantes, comme ils sont plus à risque de faire des tentatives de suicide. Les patients présentant les facteurs associés aux trajectoires d’idées suicidaires qui augmentent ou qui persistent devraient être ciblés dès le début du suivi pour bénéficierd'interventions centrées sur la prévention du suicide. Compte tenu du faible nombre de personnes faisant partie de ces trajectoires et des larges intervalles de confiance de certains facteurs, de plus vastes études sont toutefois nécessaires pour mieux caractériser qui appartient à chacun des groupes.

## Introduction

Persons with psychotic disorders are at high risk of suicide, especially in the early years after psychosis onset.^1^ Suicide rates in first-episode psychosis (FEP) are up to 18 times higher than in the general population.^2^ About a third of FEP patients experience suicidal ideation and attempts before entering services.^3^ The prevalence of suicidal ideation and attempts decreases in the first years after entering services.^3^ Suicidal ideation and attempts are major risk factors for death by suicide.^4^ Some studies have reported little overlap between FEP patients who attempted suicide at service entry and those who did so during follow-up.^5–7^ Our systematic review concluded that longitudinal studies of suicidal thoughts and behaviours in FEP were limited,^3^ with only 2 studies examining the possibility of subgroups having distinct suicidal ideation courses.^8,9^ A Danish study (*n* = 521) reported 3 trajectories of suicidal ideation over 3 years, including the year before entering a 2-year program: low decreasing (61%), frequent-stable (33%), and frequent-increasing (6%).^8^ A Spanish study (*n* = 334) also identified 3 trajectories of suicidal ideation over 2 years: non-suicidal (83%), decreasing (10%), and increasing (7%).^9^ Being a woman,^8^ being older, having a longer duration of untreated psychosis (DUP) and reduced sleep at baseline^9^ were associated with increased suicidal ideation trajectories. In the Danish study, previous suicide attempts and severe hallucinations at baseline were associated with the frequent-stable suicidal ideation trajectory. Patients in the frequent-stable and frequent-increasing subgroups were, respectively, 3 and 6 times likelier to have attempted suicide by 5 years of follow-up.

Studies considering individual variability are essential to understand the evolution of suicidal thoughts and behaviours and their predictors in FEP and guide tailored interventions for patients at risk of suicide. While suicide risk appears high in the 5 years after psychosis onset,^3^ the 2 prior trajectory studies only covered the first 2-to-3 years. Many factors were considered by these studies, but additional ones may be associated with the evolution of suicidal ideation, such as comorbidity with specific substance use disorders, cluster B personality traits, and a history of suicidal ideation.^3^

Addressing these gaps and building on the Danish^8^ and Spanish^9^ studies, we aimed to identify trajectories of suicidal ideation and their predictors over the 5-year period following the entry into early intervention services (EIS) for psychosis, while accounting for other factors potentially associated with the course of suicidal ideation. As a secondary objective, we aimed to describe and compare the distribution of suicide attempts over 5 years across identified suicidal ideation trajectories.

## Materials and Methods

### Setting and Sample

This 5-year prospective study was conducted in 2 University of Montreal-affiliated urban EIS for psychosis in Montreal, Canada: *Programme Premiers Épisodes Psychotiques* and *Clinique Jeunes Adultes Psychotiques*, serving catchments of 340,000 and 225,000, respectively. Both offer a 5-year, guidelines-based treatment course,^10^ including intensive psychiatric follow-up; recovery-oriented case management; needs-informed, developmentally appropriate individual, group and family psychosocial interventions; and supported transition to other services at the end of follow-up. Based on clinician risk assessment, standard clinical practices in cases of suicidal thoughts or behaviours included intensified support/follow-up within EIS, contact and collaboration with families, crisis centre intervention/brief stay, emergency room visits or hospitalization.

All patients admitted from 2005 to 2013 were invited to participate when the clinical team deemed them stable enough to give informed consent and participate in research interviews. The same protocol was used at both sites by the same research assistants. To be included, patients had to be aged 18–30, have a primary diagnosis of schizophrenia-spectrum disorder, affective psychotic disorder or other psychotic disorders (i.e., psychotic disorder not otherwise specified, brief psychotic disorder or delusional disorder) according to DSM-IV-TR criteria (untreated/treated for ≤1 year), and have an adequate understanding of French or English. Patients diagnosed with a psychotic disorder due to a general medical condition or with substance-induced psychosis and those with moderate to severe intellectual disability were excluded. To ensure that the study sample was representative of the patient population, we obtained approval from relevant clinical authorities and Research Ethics Boards for conducting retrospective chart reviews for *all* eligible patients, including those who could not be recruited via informed consent (e.g., due to illness severity or loss of contact, etc.).

### Assessments

#### Suicidal Ideation and Attempts

Upon admission, suicidal ideation and attempts that may have occurred during the psychotic episode that precipitated admission to the EIS were assessed categorically through interviews by a trained, experienced research assistant. Annually for 5 years, suicidal ideation and attempts within the past year were assessed using the same method. For all patients, retrospective chart reviews, which included psychiatrists’ reports and notes from case managers who followed patients very closely (including information reported by families/friends), were conducted to determine whether suicidal ideation or attempts had been reported. Coroners’ reports were requested for any patient lost to follow-up to ascertain if any fatal suicide attempt had occurred. Suicidal ideation was defined as any passive or active thoughts of suicide. Suicide attempts were defined as deliberate, self-directed, and potentially dangerous behaviours with a clear or ambivalent intent to die therefrom.

#### Potential Vulnerability Factors

Along with factors considered by previous trajectory studies^8,9^ (sex, age, occupation, functioning, alcohol use disorder, and previous suicide attempts), we included factors associated with suicidal thoughts and behaviours in FEP or that had yielded conflicting results in our systematic review^3^ (cluster B personality traits/disorder; illness severity; previous suicidal ideation; cannabis, cocaine and amphetamine use disorder assessed separately as different substances may have distinct effects on clinical and functional outcomes^11^).

Patient interviews and chart review were used to collect sociodemographic characteristics at admission, and a history of suicidal ideation and suicide attempts preceding the psychotic episode that led to entry into EIS.

At baseline, the best-estimate consensus method,^12^ involving consensus between 2 trained psychiatrists (or 1 psychiatry resident and 1 psychiatrist) based on all available information, was used to score the Alcohol Use Scale (AUS), the Drug Use Scale (DUS),^13^ the Social and Occupational Functioning Assessment Scale,^14^ and the Clinical Global Impression Scale – illness severity.^15^ The same method was used to determine DSM-IV-TR diagnosis of psychotic disorder, DSM-IV-TR diagnoses of alcohol, cannabis, cocaine or amphetamine use disorders (using all information, including AUS/DUS scores), and cluster B personality traits/disorder. All cluster B personality traits were noted and had to result in an enduring, inflexible, and pervasive pattern across a broad range of personal and social situations, leading to clinically significant distress or functional impairment.

### Statistical Analyses

Trajectory analyses included persons with no missing data on predictors *and* with at least 3 time points with valid data for suicidal ideation. Patients excluded due to missing data were compared to included patients with chi-square tests and analysis of variances (ANOVAs).

First, using a semiparametric group-based approach with a SAS-based procedure, PROC TRAJ, individual trajectories for suicidal ideation were identified.^16^ A binary logit distribution was used to identify distinctive clusters of individual trajectories over 5 years. To determine the optimal number of groups, 1- to 4-class models were fitted to the data. Based on the clinical utility of the model, the *Bayesian information criterion* (BIC), and the *Akaike information criterion* (AIC), the best-fitting model was selected. The lowest BIC and AIC values indicate the best fit. For each trajectory group, the appropriate shape (i.e., linear, quadratic, and cubic) was determined based on the same fit estimates.

Second, each person's most likely trajectory, as determined by the posterior probabilities of membership in each trajectory group generated directly by PROC TRAJ, was extracted and added as a variable to the initial dataset.

Third, we identified factors associated with membership in suicidal ideation trajectories using SPSS, v.27.0.1. We compared trajectory groups on each vulnerability factor using chi-square tests or ANOVAs. For significant factors, we performed post hoc analyses with Bonferroni correction to identify which group showed a significant difference. To avoid excluding important covariates, we integrated all factors (potentially associated with trajectory membership) simultaneously into a multivariate multinomial logistic regression. Finally, we compared the frequencies of suicide attempts by trajectory membership using chi-square tests.

## Results

Of 567 patients admitted to the 2 EIS, 382, who had valid data for at least 3 time points for suicidal ideation and selected factors, were included in our study (Supplemental Table S1 shows reasons for missing data). Sample characteristics are in Table 1. The mean age was 23.53 years; 78.01% were male; 63.52% had a schizophrenia-spectrum disorder (Supplemental Table S2 shows detailed distribution of diagnoses); and 26.96% and 8.90% had a history of suicidal ideation and attempts prior to entry, respectively.

**Table 1.** Baseline Characteristics of the Study Sample and Bivariate Comparisons by Suicidal Ideation Trajectory Membership.

	Study sample *n* = 382 (100) *n* (%)/mean (*SD*)	Trajectory group *n* (%)/mean (*SD*)			*X^2^*	*F*	*P*-value	Post hoc^c^		
		1. Low decreasing *n* = 325 (85.08)	2. Early decline, then increasing *n* = 30 (7.85)	3. Persistent *n* = 27 (7.07)				1 versus 2	1 versus 3	2 versus 3
Male	298 (78.01)	255 (78.46)	20 (66.67)	23 (85.19)	3.10		0.212			
Age	23.53 (3.61)	23.64 (2.69)	23.38 (3.10)	22.28 (2.95)		1.79	0.168			
Working or studying	142 (37.17)	121 (37.23)	14 (46.67)	7 (25.93)	2.62		0.270			
Cluster B personality traits or disorder	112 (29.32)	95 (29.23)	5 (16.67)	12 (44.44)	5.30		0.071			
Clinical illness severity—CGI^b^	4.86 (0.90)	4.84 (0.90)	5.07 (0.94)	4.85 (0.90)		1.38	0.428			
Social and occupational functioning—SOFAS^c^	33.87 (12.68)	34.14 (12.59)	32.17 (14.95)	27 (32.59)		0.480	0.619			
**Alcohol use disorder**	**72 (18.85)**	**54 (16.62)**	**7 (23.33)**	**11 (40.74)**	**9.91**		**0.007**		**<0.05**	
Cannabis use disorder	169 (44.24)	145 (44.62)	11 (36.67)	13 (48.15)	0.88		0.643			
Cocaine use disorder	21 (5.50)	17 (5.23)	3 (10.00)	1 (3.70)	1.38		0.501			
Amphetamine use disorder	50 (13.09)	46 (14.15)	2 (6.67)	2 (7.41)	2.178		0.337			
**History of suicidal thoughts and behaviours**					**22.30**		**<0.001**			
Ideation	103 (26.96)	79 (24.31)	12 (40.00)	12 (44.44)						
**Attempts**	**34 (8.90)**	**24 (7.38)**	**3 (10.00)**	**7 (25.93)**					**<0.05**	

Factors in bold are significant with a p-value < 0.05.

^a^ *Z*-test and Bonferroni correction for multiple comparisons.

^b^ Clinical Global Impression Scale—illness severity is rated on a scale from 1 to 7 with 7 indicating the highest level of severity

^c^ Social and Occupational Functioning Assessment Scale—functioning is rated using a score between 1 and 100 with 100 indicating excellent functioning.

As shown in Supplemental Table S2, compared with those excluded, included patients were likelier to have a primary schizophrenia-spectrum diagnosis vs. “other psychoses” and less likely to be working/studying and have a cocaine use disorder at admission.

Seven persons died by suicide during follow-up (see Supplemental Table S3 for details), 5 of whom were excluded for having <3 time points with valid suicidal ideation data (*n* = 4) or having missing data on selected factors (*n* = 1). The median time between admission and suicide was 11 months.

### Suicidal Ideation Trajectories

**Figure 1.**
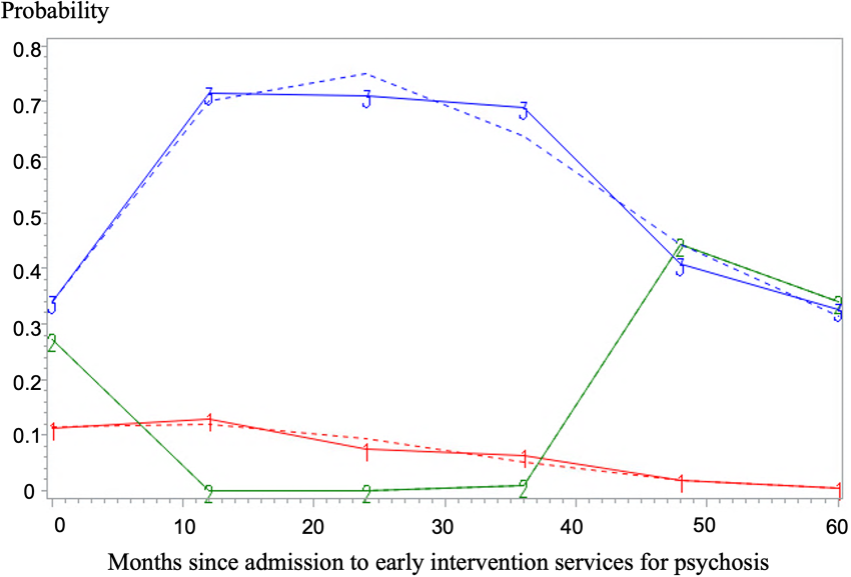
Trajectories of suicidal ideation over the 5-year follow-up. *Note*. **(**1) Low and decreasing (85.08%), (2) early decline, then increasing (7.85%), (3) persistent (7.07%). Dashed lines represent predicted values and solid lines represent observed values.

Suicidal ideation trajectories are shown in Figure 1 and fit estimates are in Table 2. The best-fitting model included 3 different classes. Although the 3-class model was associated with slightly higher BIC values than the 2-class model, the 3-class model allowed the detection of the *early decline, then increasing suicidal ideation* trajectory, which deserves examination for its clinical implications. In contrast, the 4-class model only added a trajectory very similar to the *low and decreasing* one and had higher BIC and AIC values.

**Table 2.** Fit Estimates for 1-to-4 Class Solutions of Suicidal Ideation Trajectories.

	Fit estimates^a^		
	BIC (*n* = 2018)	aBIC^b^ (*n* = 382)	AIC
One class	757.13	753.80	745.91
Two classes^c^	744.10	736.61	718.86
Three classes	752.00	740.64	712.73
Four classes	767.96	752.15	714.67

^a^ Lowest Bayesian information criterion (BIC) and Akaike information criterion (AIC) values indicate the best fit.

^b^ Sample-size-adjusted BIC (aBIC).

^c^ The two-class model included, with slightly larger proportions than the 3 class-model, *low and decreasing* and *persistent* suicidal ideation trajectories.

The first group, including most patients (*n* = 325, 85.08%), followed a trajectory of *low and decreasing* probability of presenting suicidal ideation. The second group (*n* = 30, 7.85%), followed a trajectory of *early decline, then increasing suicidal ideation*. The probability of presenting suicidal ideation at admission was moderately high for this group (≈30%) followed by an absence of suicidal ideation until the last 2 years of follow-up, when the probability increased again to ≈45%. The third group (*n* = 27, 7.07%) followed a trajectory of *persistent suicidal ideation*, wherein the probability of having suicidal ideation at entry was moderately high (≈35%) and increased to ≈70% between the first and third year of follow-up and then decreased in the last follow-up year to ≈40%.

### Factors Associated With Trajectory Membership

#### Bivariate Analyses

As presented in Table 1, patients in the *persistent suicidal ideation* trajectory were likelier to have an alcohol use disorder at admission and to have attempted suicide prior to admission than those in the *low and decreasing suicidal ideation* trajectory.

#### Multinomial Logistic Regression

The *low and decreasing* trajectory group was the reference category. History of suicidal ideation before admission and a baseline cocaine use disorder significantly increased the odds of belonging to the *early decline, then increasing suicidal ideation* trajectory by 2.85 [95% CI, 1.23 to 6.63] and 6.78 times [95% CI, 1.08 to 42.75], respectively, compared to the *low and decreasing* group (Table 3). History of suicidal ideation and suicide attempts were the factors most strongly associated with *persistent suicidal ideation* compared with the *low and decreasing suicidal ideation* trajectory. Alcohol use disorder at admission was also associated with the *persistent suicidal ideation* trajectory as compared to the *low and decreasing suicidal ideation* trajectory.

**Table 3.** Multinomial Logistic Regression: Factors Associated With Suicidal Ideation Trajectory Membership.

	*B*	*SE*	*P*-value	OR	95% CI
Early decline and increasing suicidal ideation^b^					
Male	−0.712	0.453	0.116	0.491	[0.202 to 1.192]
Age	−0.050	0.059	0.389	0.951	[0.848 to 1.066]
Working or studying	−0.448	0.416	0.281	1.565	[0.693 to 3.533]
Cluster B personality traits or disorder	−0.892	0.553	0.107	0.410	[0.139 to 1.213]
Clinical illness severity—CGI^c^	0.287	0.319	0.367	1.333	[0.714 to 2.489]
Social and Occupational Functioning—SOFAS^d^	−0.004	0.023	0.858	0.996	[0.952 to 1.042]
Alcohol use disorder	0.629	0.533	0.238	1.876	[0.660 to 5.328]
Cannabis use disorder	−0.170	0.478	0.722	0.844	[0.330 to 2.155]
**Cocaine use disorder**	**1.914**	**0.939**	**0.042**	**6.783**	**[1.076 to 42.753]**
Amphetamine use disorder	−1.677	1.028	0.103	0.187	[0.025 to 1.402]
History of suicidal thoughts and behaviours (reference category = none)					
**Ideation**	**1.047**	**0.431**	**0.015**	**2.849**	**[1.225 to 6.629]**
Attempts	0.816	0.724	0.260	2.262	[0.547 to 9.349]
Persistent suicidal ideation^a,d^					
Male	0.196	0.624	0.753	1.217	[0.358 to 4.135]
Age	−0.109	0.065	0.096	0.897	[0.789 to 1.019]
Working or studying	−0.363	0.496	0.464	0.695	[0.263 to 1.840]
Cluster B personality traits or disorder	0.393	0.476	0.410	1.481	[0.582 to 3.768]
Clinical illness severity—CGI^c^	−0.183	0.328	0.577	0.833	[0.437 to 1.585]
Social and Occupational Functioning—SOFAS^d^	−0.009	0.024	0.706	0.991	[0.944 to 1.039]
**Alcohol use disorder**	**1.289**	**0.487**	**0.008**	**3.628**	**[1.397 to 9.424]**
Cannabis use disorder	−0.163	0.489	0.739	0.850	[0.326 to 2.218]
Cocaine use disorder	0.165	1.271	0.896	1.180	[0.098 to 14.248]
Amphetamine use disorder	−1.543	0.931	0.097	0.214	[0.034 to 1.326]
**History of suicidal thoughts and behaviours (reference category = none)**					
**Ideation**	**1.466**	**0.489**	**0.003**	**4.332**	**[1.662 to 11.292]**
**Attempts**	**2.102**	**0.627**	**<0.001**	**8.181**	**[2.393 to 27.973]**

Factors in bold are significant with a p-value <‌‌ 0.05.

^a^ Reference category = *low and decreasing suicidal ideation* trajectory.

^b^ Clinical global impression scale—illness severity is rated on a scale from 1 to 7 with 7 indicating the highest level of severity.

^c^ Social and occupational functioning assessment scale—functioning is rated using a score between 1 and 100 with 100 indicating excellent functioning.

^d^ The reference category was changed to compare persons in the *early decline, then increasing suicidal ideation* trajectory and those in the *persistent suicidal ideation* trajectory. Except for a tendency that indicates that patients with Cluster B personality traits or disorder were more likely to be in the *persistent suicidal ideation* trajectory compared to the *early decline, then increasing* 1 (OR = 3.613 [95% CI, 0.927 to 14.079], *P* = 0.064), no significant differences were detected between these trajectory groups.

The reference category was changed to compare the *early decline, then increasing* and the *persistent suicidal ideation* trajectory groups. No significant differences were identified, although there was a tendency for persons with cluster B personality traits/disorder being likelier to belong to the *persistent suicidal ideation* versus the *early decline, then increasing* trajectory (OR = 3.613 [95% CI, 0.927 to 14.079], *P* = 0.064).

Sensitivity analyses were conducted by including 5 of the 7 persons who died by suicide (those who had data for potential vulnerability factors) in the *persistent suicidal ideation* trajectory. Results for factors associated with membership in the *persistent* trajectory remained unchanged.

### Frequency of Suicide Attempts by Trajectory Membership

Twenty-five out of 382 patients (6.54%) attempted suicide at least once during follow-up, including 6 (1.57%) who reported at least 1 attempt in the past year during 2 of the 5-yearly assessments, and 1 person (0.26%) at 3 assessments.

Except for the fourth year, in which no patient attempted suicide, the frequency of suicide attempts differed significantly by trajectory membership in all years of follow-up (Table 4). At admission and for the following 3 years, a greater proportion attempted suicide in the *persistent suicidal ideation* trajectory group than those in the other 2 trajectories.

**Table 4.** Distribution of Suicidal Ideation and Suicide Attempts for Each Year of Follow-up by Trajectory Membership.

Time point	Total sample *n* = 382 (100)	Trajectory group			*X^2^*	*P*-value
		1. Low decreasing *n* = 325 (85.08)	2. Early decline, then increasing *n* = 30 (7.85)	3. Persistent *n* = 27 (7.07)		
**Suicidal ideation**					
Admission	57 (15.24)	39 (12.26)	8 (26.67)	10 (34.86)	16.07	<0.001
Year 1	60 (16.30)	42 (13.42)	0 (0)	18 (72.00)	64.58	<0.001
Year 2	43 (12.01)	23 (7.54)	0 (0)	20 (83.33)	125.24	<0.001
Year 3	36 (10.65)	18 (6.25)	0 (0)	18 (81.82)	126.29	<0.001
Year 4	32 (10.19)	0 (0)	22 (75.86)	10 (52.63)	204.23	<0.001
Year 5	19 (7.17)	0 (0)	13 (48.15)	6 (37.50)	107.38	<0.001
**Suicide attempts**					
Admission	5 (1.32)	1 (0.31)	1 (3.33)	3 (11.54)	24.25	<0.001
Year 1	6 (1.63)	2 (0.64)	0 (0)	4 (16.00)	34.60	<0.001
Year 2	15 (4.18)	6 (1.96)	0 (0)	9 (37.50)^a^	71.58	<0.001
Year 3	4 (1.18)	1 (0.35)	0 (0)	3 (14.29)^a^	32.90	<0.001
Year 4	0 (0)	0 (0)	0 (0)	0 (0)	—	—
Year 5	3 (1.13)	0 (0)	2 (7.41)	1 (6.25)	15.79	<0.001

^a^ One patient died as a result of suicide attempt.

Towards the end of follow-up, while no patients in the *low and decreasing suicidal ideation* trajectory attempted suicide, 7.41% and 6.25% of patients in the *early decline, then increasing suicidal ideation* and the *persistent suicidal ideation* trajectories, respectively, reported suicide attempts. Two patients, both in the *persistent suicidal ideation* trajectory, died by suicide at 2 and 3 years of follow-up.

## Discussion

Our study identified 3 distinct suicidal ideation trajectories over a 5-year follow-up of FEP patients: *low and decreasing* (*n* = 325, 85.08%); *early decline, then increasing* (*n* = 30, 7.85%); and *persistent suicidal ideation* (*n* = 27, 7.07%).

Like for the 2 other studies of suicidal ideation trajectories in FEP,^8,9^ we identified a trajectory including most patients characterized by no/low and decreasing suicidal ideation. Nonetheless, some showed persistent or increasing suicidal ideation. Seven percent followed a trajectory of suicidal ideation that persisted in a fluctuating pattern, which is lower than, but consistent, with the frequent and stable suicidal ideation trajectory in the Danish study (*n* = 172, 33%).

Consistent with the Danish study,^8^ we found that patients with suicidal ideation and suicide attempts before entering services were at higher risk of following the *persistent suicidal ideation* trajectory. A greater proportion in the *persistent ideation* trajectory, as well as both persons who died by suicide after 1 year of follow-up, attempted suicide during the 5-year follow-up, suggesting that, as in the general population, suicidal ideation is a risk factor for attempts in FEP patients.^3,8,17^

Alcohol use disorder (the most prevalent substance use disorder in Canada^18,19^) is a major risk factor for suicide in the general population and alcohol is the most frequently identified substance in suicide decedents.^20,21^ Yet, previous studies on the association between alcohol use disorder and suicidal risk in FEP yielded inconsistent results.^3^ We contend that alcohol use may not have emerged as a risk factor when considering only group averages and may be pertinent for FEP patient subgroups with less favourable suicidal courses. In our study, alcohol use disorder was associated with the *persistent suicidal ideation* trajectory.

Five of the 7 persons who died by suicide had a cannabis use disorder. Of these, 4 had a concurrent alcohol use disorder, 3 had 3 substance use disorders (cannabis, alcohol, and amphetamine) and 2 had 4 substance use disorders (cannabis, alcohol, amphetamine, and cocaine). These descriptive results highlight that alcohol and polysubstance use may be important to consider in suicide in FEP. Given that substance use is strongly associated with both suicidal risk and psychotic experiences,^22^ further studies should assess the role of concurrent substance use in the association between psychosis and suicide in FEP.

In the general population, personality disorders, particularly borderline personality disorder, have been associated with increased suicidal risk.^23^ Although not statistically significant, only a trend (*p* = 0.064), our findings suggest that FEP patients with cluster B personality traits/disorder may be more likely to follow a trajectory of *persistent* ideation compared to *early decline, then increasing* trajectory.

While the other 2 trajectory studies in FEP also identified a trajectory of increasing suicidal ideation,^8,9^ ours (7.85%) is slightly different as the increase was preceded by an early decline and absence of suicidal ideation. It is unclear whether the estimation of linear, quadratic, and cubic terms (allowing detection of curved trajectories), the different study periods and countries, or the different measures of suicidal ideation explain these variations.

Factors associated with the increasing suicidal ideation trajectory differ between FEP studies. In our study, we found that persons with prior suicidal ideation and cocaine use disorder at admission were at greater risk for following such trajectories, although for cocaine use disorder, this relates to only 3 people and warrants further studies. Except for sex and age, no factors were assessed by even 2 studies, making it impossible to examine whether the results converge. Nevertheless, factors associated with increased suicidal ideation (i.e., longer DUP, reduced sleep, prior suicidal ideation, and cocaine use disorder) in the 3 trajectory studies (Danish, Spanish and ours), have all been associated with poorer psychosis outcomes.^24–26^ Thus, further studies should assess whether the increase in suicidal ideation for some patients is due to less favourable evolution and less treatment responsiveness.

The early decline in the trajectory of increasing suicidal ideation may be due to feeling supported, response to treatment, a better understanding of their psychotic experience, etc. Similarly, the increase at the end of follow-up may be explained by various factors related to this period: changes in the therapeutic relationship, transition between services and lack of continuity in care,^27^ continued disability, relapse,^28^ etc. Further studies, including qualitative ones, are needed to understand this increase, and more generally, how factors, such as stigma,^29^ perceived control and the understanding and meaning given to the psychotic experience, may influence both recovery^30–32^ and suicide risk in FEP.

## Implications

Despite the decrease in suicidal ideation for most patients, some presented persistent and increasing suicidal ideation, stressing the need for ongoing assessment and management of suicide risk in FEP.

The first year of follow-up was characterized by the majority of deaths by suicide (4 out of 7), a marked increase in suicidal ideation for the *persistent* trajectory and a decline in suicidal ideation for the *early decline, then increasing* trajectory, suggesting that the beginning of follow-up is particularly important for suicide prevention.

Suicide-focused psychosocial interventions (notably cognitive behavioural therapy and supportive interventions by a case manager) were associated with decreased suicidal thoughts and behaviours in FEP in a meta-analysis.^33^ Another recent study showed that a decrease in suicidal ideation in FEP was predicted by the number of psychotherapy sessions.^34^ Our findings underline that such interventions should be implemented from early on in follow-ups.

Patients with a history of suicidal thoughts and behaviours and with substance use disorder at admission could be targeted early for these interventions and assessed frequently for suicidal risk. As outlined in the guidelines, treatment for these comorbid disorders should also be integrated into EIS early on.^10^ Persons with persistent suicidal ideation may require attention throughout follow-up given their increased risk of attempting suicide.

### Strengths and Limitations

This is the first study to identify suicidal ideation trajectories throughout the 5-year postonset critical period in FEP. Considering individual variability allowed us to identify different evolution patterns. Ours is also the first study to examine whether suicidal ideation trajectories were differentially associated with suicide attempts during follow-up, which EIS seek to prevent. We included a range of potential factors, guided by our systematic review and prior studies.

Nonetheless, our study has certain limitations. We excluded persons with <3 time points of valid suicidal ideation data or missing data on chosen factors. Although several sociodemographic and clinical characteristics were similar between excluded patients and the study sample, included patients were likelier to be unemployed/not studying and to have a schizophrenia-spectrum diagnosis. Since these characteristics have been associated with poorer long-term outcomes,^35,36^ our study sample may have had a worse prognosis than the excluded patients. Included patients were also less likely to have a cocaine use disorder, which was associated with the *early decline, then increasing* trajectory*.* A stronger association may have been observed had more of these patients been included. Excluding 5 of the 7 patients who died by suicide resulted in excluding individuals with severe suicidal risk. Nevertheless, their inclusion in the *persistent suicidal ideation* trajectory did not change the results. Furthermore, our study is fairly representative of suicidal risk in FEP as our sample was drawn from all treated FEP cases in 2 catchment areas.

Two trajectories included only 7% of participants, which may have limited our power to identify predictors. Some significant associations were driven by very few persons (e.g., cocaine use), as reflected by the wide confidence intervals. Nevertheless, the identified predictors had moderate to large effect sizes. The low number of suicide attempts precluded identification of trajectories of suicide attempts or associated factors. However, we went beyond describing trajectories of suicide attempts by comparing the distribution of attempts across the 3 suicidal ideation trajectories and showing that more patients with persistent suicidal ideation attempted suicide.

We included factors measured at admission, although some would have evolved during follow-up (e.g., symptoms, functioning, and substance use). Considering these factors longitudinally would allow for a better understanding of how their evolution is associated with changes in suicidal risk, with significant implications for assessment and intervention.

Potentially important factors, such as depressive symptoms, impulsivity, and DUP,^37^ were not considered. Relying on annual, yes/no assessments, our measure of suicidal ideation and attempts did not account for frequency or severity. Informed by our study, we propose several valuable future research directions (Supplemental Table S4).

## Conclusion

Our study extends previous trajectory studies of suicidal ideation to a longer 5-year period. Our findings underscore the importance of suicide prevention interventions being offered from the beginning of FEP follow-up and close monitoring, particularly of those with a history of suicidal thoughts and behaviours, alcohol use disorder and those who persistently report suicidal ideation. Further research is needed to better characterize who belongs in each group and study group-specific prevention strategies.

## Supplemental Material

sj-docx-1-cpa-10.1177_07067437231167387 - Supplemental material for Heterogeneity in the Course of Suicidal Ideation and its Relation to Suicide Attempts in First-Episode Psychosis: A 5-Year Prospective Study Click here for additional data file. Supplemental material, sj-docx-1-cpa-10.1177_07067437231167387 for Heterogeneity in the Course of Suicidal Ideation and its Relation to Suicide Attempts in First-Episode Psychosis: A 5-Year Prospective Study by Roxanne Sicotte and Srividya N. Iyer, Éric Lacourse, Jean R. Séguin, Amal Abdel-Baki in The Canadian Journal of Psychiatry

sj-docx-2-cpa-10.1177_07067437231167387 - Supplemental material for Heterogeneity in the Course of Suicidal Ideation and its Relation to Suicide Attempts in First-Episode Psychosis: A 5-Year Prospective Study Click here for additional data file. Supplemental material, sj-docx-2-cpa-10.1177_07067437231167387 for Heterogeneity in the Course of Suicidal Ideation and its Relation to Suicide Attempts in First-Episode Psychosis: A 5-Year Prospective Study by Roxanne Sicotte and Srividya N. Iyer, Éric Lacourse, Jean R. Séguin, Amal Abdel-Baki in The Canadian Journal of Psychiatry

sj-docx-3-cpa-10.1177_07067437231167387 - Supplemental material for Heterogeneity in the Course of Suicidal Ideation and its Relation to Suicide Attempts in First-Episode Psychosis: A 5-Year Prospective Study Click here for additional data file. Supplemental material, sj-docx-3-cpa-10.1177_07067437231167387 for Heterogeneity in the Course of Suicidal Ideation and its Relation to Suicide Attempts in First-Episode Psychosis: A 5-Year Prospective Study by Roxanne Sicotte and Srividya N. Iyer, Éric Lacourse, Jean R. Séguin, Amal Abdel-Baki in The Canadian Journal of Psychiatry

sj-docx-4-cpa-10.1177_07067437231167387 - Supplemental material for Heterogeneity in the Course of Suicidal Ideation and its Relation to Suicide Attempts in First-Episode Psychosis: A 5-Year Prospective Study Click here for additional data file. Supplemental material, sj-docx-4-cpa-10.1177_07067437231167387 for Heterogeneity in the Course of Suicidal Ideation and its Relation to Suicide Attempts in First-Episode Psychosis: A 5-Year Prospective Study by Roxanne Sicotte and Srividya N. Iyer, Éric Lacourse, Jean R. Séguin, Amal Abdel-Baki in The Canadian Journal of Psychiatry
