# Supplementary material for: Heterogeneity in the Course of Suicidal Ideation and its Relation to Suicide Attempts in First-Episode Psychosis: A 5-Year Prospective Study
Source: Can J Psychiatry. 2023 Apr 18;68(11):850–9. doi: 10.1177/07067437231167387 (PMC10590090; doi:10.1177/07067437231167387)
Supplement: sj-docx-1-cpa-10.1177_07067437231167387 - Supplemental material for Heterogeneity in the Course of Suicidal Ideation and its Relation to Suicide Attempts in First-Episode Psychosis: A 5-Year Prospective Study [file sj-docx-1-cpa-10.1177_07067437231167387.docx]

Heterogeneity in the course of suicidal ideation and its relation to suicide attempts in first-episode psychosis: a five-year prospective study

Journal: The Canadian Journal of Psychiatry

Authors: Roxanne Sicotte, Srividya N. Iyer, Éric Lacourse, Jean R. Séguin, Amal Abdel-Baki

Corresponding author: Amal Abdel-Baki (amal.abdel-baki@umontreal.ca)

Research Center Centre Hospitalier de l'Université de Montréal (CRCHUM), Montréal, Québec, Canada

Department of Psychiatry and Addiction, Faculty of Medicine, Montréal, Québec, Canada

**Table S1. Reasons for exclusion (n=185)**

| Time point | Reasons for exclusion | n (%) |
| --- | --- | --- |
| Baseline | Missing data on selected predictors | 27 (14.59) |
| Year 1 | Lost to follow-up | 54 (29.19) |
|  | Transferred to another program | 23 (12.43) |
|  | Moved to another province in Canada or another Country | 21 (11.35) |
|  | Has been incarcerated | 1 (0.54) |
|  | Died by suicide | 4 (2.16) |
| Year 2 | Lost to follow-up | 26 (14.05) |
|  | Transferred to another program | 13 (7.03) |
|  | Moved to another province in Canada or another Country | 5 (2.70) |
|  | Died due to natural cause | 1 (0.54) |
| All time points combined | Followed for at least three time points but data on suicidal ideation was missing | 10 (5.41) |
|  | **TOTAL** | **185 (100)** |
