# Supplementary material for: Heterogeneity in the Course of Suicidal Ideation and its Relation to Suicide Attempts in First-Episode Psychosis: A 5-Year Prospective Study
Source: Can J Psychiatry. 2023 Apr 18;68(11):850–9. doi: 10.1177/07067437231167387 (PMC10590090; doi:10.1177/07067437231167387)
Supplement: sj-docx-2-cpa-10.1177_07067437231167387 - Supplemental material for Heterogeneity in the Course of Suicidal Ideation and its Relation to Suicide Attempts in First-Episode Psychosis: A 5-Year Prospective Study [file sj-docx-2-cpa-10.1177_07067437231167387.docx]

Heterogeneity in the course of suicidal ideation and its relation to suicide attempts in first-episode psychosis: a five-year prospective study

Journal: The Canadian Journal of Psychiatry

Authors: Roxanne Sicotte, Srividya N. Iyer, Éric Lacourse, Jean R. Séguin, Amal Abdel-Baki

Corresponding author: Amal Abdel-Baki (amal.abdel-baki@umontreal.ca)

Research Center Centre Hospitalier de l'Université de Montréal (CRCHUM), Montréal, Québec, Canada

Department of Psychiatry and Addiction, Faculty of Medicine, Montréal, Québec, Canada

**Table S2. Comparison of study sample and patients excluded because of missing data on suicidal ideation and potential vulnerability factors**

|  | Total  (n=567)  n (%)/M (SD) | Patients excluded (n=185)  n (%)/M (SD) | Study sample  (n=382)  n (%)/M (SD) | *p*-value |
| --- | --- | --- | --- | --- |
| Male | 429 (75.66) | 131 (70.81) | 298 (78.01) | .061 |
| Age at admission | 23.59 (3.55) | 23.74 (3.43) | 23.53 (3.61) | .473 |
| Working or studying | **229 (40.46)** | **87 (47.28)** | **142 (37.17)** | **.022** |
| Principal diagnosis |  |  |  | **<.001^a^** |
| Schizophrenia spectrum disorder | **330 (58.20)** | **88 (47.57)** | **242 (63.35)** |  |
| Schizophrenia | 231 (40.74) | 60 (32.43) | 171 (44.76) |  |
| Schizoaffective disorder | 59 (10.41) | 9 (4.87) | 50 (13.09) |  |
| Schizophreniform disorder | 40 (7.05) | 19 (10.27) | 21 (5.50) |  |
| Affective psychosis | **157 (27.69)** | **57 (30.81)** | **100 (26.18)** |  |
| Bipolar I disorder with psychotic features | 143 (25.22) | 53 (28.65) | 90 (23.56) |  |
| Major depressive episode with psychotic features | 14 (2.47) | 4 (2.16) | 10 (2.62) |  |
| Other psychoses | **80 (14.11)** | **40 (21.62)** | **40 (10.47)** |  |
| Psychotic disorder not otherwise specified | 70 (12.35) | 34 (18.38) | 36 (9.42) |  |
| Brief psychotic disorder | 8 (1.41) | 5 (2.70) | 3 (0.79) |  |
| Delusional disorder | 2 (0.35) | 1 (0.54) | 1 (0.26) |  |
| Cluster B personality traits or disorder | 165 (29.52) | 53 (29.94) | 112 (29.32) | .880 |
| Clinical illness severity-CGI^b^ | 4.82 (0.92) | 4.73 (0.96) | 4.86 (0.90) | .125 |
| Social and Occupational Functioning- SOFAS^c^ | 34.37 (13.14) | 35.38 (14.04) | 33.87 (12.68) | .202 |
| Alcohol use disorder | 113 (20.00) | 41 (22.40) | 72 (18.85) | .323 |
| Cannabis use disorder | 254 (44.88) | 85 (46.20) | 169 (44.24) | .661 |
| Cocaine use disorder | **40 (7.08)** | **19 (10.38)** | **21 (5.50)** | **.034** |
| Amphetamine use disorder | 80 (14.16) | 30 (16.39) | 50 (13.09) | .292 |
| Suicidal ideation at admission | 84 (15.53) | 27 (16.17) | 57 (15.24) | .783 |
| Suicide attempt at admission | 9 (1.64) | 4 (2.35) | 5 (1.32) | .380 |
| History of suicidal thoughts and behaviours |  |  |  |  |
| Ideation | 146 (27.70) | 43 (29.66) | 103 (26.96) | .537 |
| Attempts | 44 (8.29) | 10 (6.71) | 34 (8.90) | .411 |
| Died over the follow-up |  |  |  | .088 |
| Died by suicide | 7 (1.23) | 5 (2.70) | 2 (0.52) |  |
| Died due to natural causes | 3 (0.53) | 1 (0.54) | 2 (0.52) |  |

^a^ Post hoc analyses (Z-test and adjusted significance value with Bonferroni correction) indicate that persons with a schizophrenia spectrum disorder are more likely to be included in our study compared to those with a diagnosis of “other psychoses” (p<0.05).

^b^ Clinical Global Impression Scale – illness severity is rated on a scale from 1-7 with 7 indicating the highest level of severity

^c^ Social and Occupational Functioning Assessment Scale – functioning is rated using a score between 1-100 with 100 indicating excellent functioning
