# Supplementary material for: Heterogeneity in the Course of Suicidal Ideation and its Relation to Suicide Attempts in First-Episode Psychosis: A 5-Year Prospective Study
Source: Can J Psychiatry. 2023 Apr 18;68(11):850–9. doi: 10.1177/07067437231167387 (PMC10590090; doi:10.1177/07067437231167387)
Supplement: sj-docx-3-cpa-10.1177_07067437231167387 - Supplemental material for Heterogeneity in the Course of Suicidal Ideation and its Relation to Suicide Attempts in First-Episode Psychosis: A 5-Year Prospective Study [file sj-docx-3-cpa-10.1177_07067437231167387.docx]

Heterogeneity in the course of suicidal ideation and its relation to suicide attempts in first-episode psychosis: a five-year prospective study

Journal: The Canadian Journal of Psychiatry

Authors: Roxanne Sicotte, Srividya N. Iyer, Éric Lacourse, Jean R. Séguin, Amal Abdel-Baki

Corresponding author: Amal Abdel-Baki (amal.abdel-baki@umontreal.ca)

Research Center Centre Hospitalier de l'Université de Montréal (CRCHUM), Montréal, Québec, Canada

Department of Psychiatry and Addiction, Faculty of Medicine, Montréal, Québec, Canada

**Table S3. Characteristics of persons who died by suicide during follow-up**

|  | Patients who died by suicide  (n=7)  n (%)/M (SD) |
| --- | --- |
| Male | 5 (71.43) |
| Age | 22.50 (2.55) |
| Working or studying | 3 (42.86) |
| Principal diagnosis |  |
| Schizophrenia | 5 (71.43) |
| Bipolar I disorder with psychotic features | 2 (28.57) |
| Cluster B personality traits or disorder | 3 (50.00) |
| Clinical illness severity-CGI^a^ | 4.86 (0.90) |
| Social and Occupational Functioning- SOFAS^b^ | 35.71 (15.12) |
| Cannabis use disorder | 5 (71.43) |
| Alcohol use disorder | 4 (57.14)^c^ |
| Amphetamine use disorder | 3 (42.86)^d^ |
| Cocaine use disorder | 2 (28.57)^e^ |
| Suicidal ideation at admission | 2 (33.33) |
| Suicide attempt at admission | 1 (16.67) |
| Previous history of suicidal thoughts and behaviours |  |
| Ideation | 1 (20.00) |
| Attempts | 1 (16.67) |
| Time to suicide (months; range from 21 days to 46 months) | 16.14 (15.41) |
| Death by suicide – year 1 | 4 (57.14) |
| Death by suicide – year 2 | 1 (14.29) |
| Death by suicide – year 3 | 1 (14.29) |
| Death by suicide – year 4 | 1 (14.29) |
| Death by suicide – year 5 | 0 (0) |

^a^ Clinical Global Impression Scale

^b^ Social and Occupational Functioning Assessment Scale

^c^ These four patients also had a cannabis use disorder

^d^ These three patients also had a cannabis use disorder and an alcohol use disorder

^e^ These two patients also had a cannabis use disorder, an alcohol use disorder and an amphetamine use disorder
