# Supplementary material for: Heterogeneity in the Course of Suicidal Ideation and its Relation to Suicide Attempts in First-Episode Psychosis: A 5-Year Prospective Study
Source: Can J Psychiatry. 2023 Apr 18;68(11):850–9. doi: 10.1177/07067437231167387 (PMC10590090; doi:10.1177/07067437231167387)
Supplement: sj-docx-4-cpa-10.1177_07067437231167387 - Supplemental material for Heterogeneity in the Course of Suicidal Ideation and its Relation to Suicide Attempts in First-Episode Psychosis: A 5-Year Prospective Study [file sj-docx-4-cpa-10.1177_07067437231167387.docx]

Heterogeneity in the course of suicidal ideation and its relation to suicide attempts in first-episode psychosis: a five-year prospective study

Journal: The Canadian Journal of Psychiatry

Authors: Roxanne Sicotte, Srividya N. Iyer, Éric Lacourse, Jean R. Séguin, Amal Abdel-Baki

Corresponding author: Amal Abdel-Baki (amal.abdel-baki@umontreal.ca)

Research Center Centre Hospitalier de l'Université de Montréal (CRCHUM), Montréal, Québec, Canada

Department of Psychiatry and Addiction, Faculty of Medicine, Montréal, Québec, Canada

**Table S4. Future research directions**

| What should be studied? | Time-varying and potentially proximal risk factors for suicidal ideation and suicide attempts, such as psychotic and depressive symptoms. |
| --- | --- |
|  | Detailed examination of associations between Cluster B personality traits/disorder and suicidal thoughts and behaviours in FEP as our results suggested a trend in this regard and this factor has been rarely studied in persons with FEP |
|  | The effect of specific types of interventions, pharmacological treatment, and hospitalizations should be assessed as these may influence suicidal risk^1;2^. |
|  | The association between different trajectories and subsequent deaths by suicide. |
|  | The effect of interventions targeting modifiable factors on suicidal risk. |
| How should it be studied? | Longitudinal studies assessing most variables at all time points in larger samples. |
|  | To provide a more accurate picture of the fluctuation in suicidal risk, further studies should include more frequent assessments using a scale designed specifically to assess suicidal thoughts and behaviours. |
|  | Suicide deaths are rare events, making them difficult to study, especially if EIS does reduce their prevalence. To better understand what is associated with suicide deaths, future studies should use all available data from all sources up to the point of death by suicide, combine data from multiple EIS, and use specific methodologies for these situations such as psychological autopsies. |
|  | Qualitative studies could unpack what underlies persistent or increasing suicidal ideation and suicide attempts. |

1. Pelizza L, Maestri D, Leuci E et al. 2021. Individual psychotherapy can reduce suicidal ideation in first episode psychosis: Further findings from the 2-year follow-up of the 'parma early psychosis' programme. Clin Psychol Psychother.

2. Pompili M. 2020. Adding suicide prevention to the triple advantages of injectable long-acting second-generation antipsychotics. Frontiers in Psychiatry. 10.
